# An online survey investigating the information needs of adolescent idiopathic scoliosis patients and their families: preliminary results

**DOI:** 10.1186/1748-7161-8-S1-O54

**Published:** 2013-06-03

**Authors:** S Wellburn, J Bettany-Saltikov, S Hamilton

**Affiliations:** 1 Teesside University, Middlesbrough, UK

## Background

Government policy documents have identified the need for good information for patients; a notion supported throughout the literature, in particular the need for written information. Despite a reported wealth of leaflets and booklets being available providing such information, the failure to address the information requirements of patients and their families is consistently noted in the literature. A preliminary review of the literature regarding scoliosis did not find any studies that have investigated service users perspectives of what should be provided as written information. This would suggest that the information materials produced by scoliosis centres are based on what health professionals think the patients need to know rather than what they actually want to know.

## Aim

To investigate the information needs of AIS patients and their families, from a national perspective.

## Methods

A survey was constructed that consisted of 18 questions in total; these comprised pre-suggested responses for respondents to select and open-ended text boxes to illicit respondents perspectives. A link to the survey was attached to the Scoliosis Association UK Web site in order that visitors to the site could complete the survey.

## Results

Twenty-two responses were received from 18 different postcode regions. Analysis showed a mean age of 12 years old, 82% of whom were female with 77% completing the survey with their parents, 23% on their own. The most common curve type was thoracic (45%) and over 80% had curves greater than 30 degrees at first diagnosis. Variability between centres the in the quality of information received by respondents was suggested by respondents ratings of materials and information received.

## Conclusion

Patients, and their carers, identified a need for additional information, and that verbal information was not sufficient as a standalone model. It was advocated that information should be made accessible between GP referral and first consultation with the consultant. It was recommended that in all formats information provided should be user friendly and in plain language. The opportunity to communicate with other AIS patients was suggested to be a potentially valuable resource.
